# Ablation of liver *Fxr* results in an increased colonic mucus barrier in mice

**DOI:** 10.1016/j.jhepr.2021.100344

**Published:** 2021-08-04

**Authors:** Noortje Ijssennagger, Kristel S. van Rooijen, Stefanía Magnúsdóttir, José M. Ramos Pittol, Ellen C.L. Willemsen, Marcel R. de Zoete, Matthijs J.D. Baars, Paul B. Stege, Carolina Colliva, Roberto Pellicciari, Sameh A. Youssef, Alain de Bruin, Yvonne Vercoulen, Folkert Kuipers, Saskia W.C. van Mil

**Affiliations:** 1 Department of Molecular Cancer Research, Center for Molecular Medicine, University Medical Center Utrecht, Utrecht, The Netherlands; 2 Institute of Biochemistry, University of Innsbruck, Innsbruck, Austria; 3 Department of Medical Microbiology, University Medical Center Utrecht, Utrecht, The Netherlands; 4 TES Pharma S.r.l., Perugia, Italy; 5 Non-Clinical Safety, Department of Pathology, Janssen Pharmaceutica Research and Development, Beerse, Belgium; 6 Departments of Pediatrics and Laboratory Medicine, University of Groningen, University Medical Center Groningen, Groningen, the Netherlands; 7 Department of Biomolecular Health Sciences, Faculty of Veterinary Medicine, Utrecht University, Utrecht, the Netherlands

**Keywords:** Farnesoid X receptor, Liver-specific *Fxr*-KO mouse, Intestine-specific *Fxr*-KO mouse, Colon, Gut microbiome, Mucus layer, Liver–gut axis, BAs, bile acids, DSS, dextran sodium sulfate, Fgfr4, fibroblast growth factor receptor 4, FITC, fluorescein isothiocyanate, fpkm, fragments per kilobase of transcript per million mapped reads, *Fxr*, farnesoid X receptor, *Fxr*-intKO, intestine-specific *Fxr* knockout, *Fxr*-livKO, liver-specific *Fxr* knockout, *Fxr*-totKO, whole body *Fxr* knockout, GO, Gene Ontology, HID, high-iron diamine, IBD, inflammatory bowel disease, KEGG, Kyoto Encyclopedia of Genes and Genomes, RT qPCR, real-time quantitative PCR

## Abstract

**Background & Aims:**

The interorgan crosstalk between the liver and the intestine has been the focus of intense research. Key in this crosstalk are bile acids, which are secreted from the liver into the intestine, interact with the microbiome, and upon absorption reach back to the liver. The bile acid-activated farnesoid X receptor (*Fxr*) is involved in the gut-to-liver axis. However, liver-to-gut communication and the roles of bile acids and *Fxr* remain elusive. Herein, we aim to get a better understanding of *Fxr*-mediated liver-to-gut communication, particularly in colon functioning.

**Methods:**

Fxr floxed/floxed mice were crossed with cre-expressing mice to yield *Fxr* ablation in the intestine (*Fxr*-intKO), liver (*Fxr*-livKO), or total body (*Fxr*-totKO). The effects on colonic gene expression (RNA sequencing), the microbiome (16S sequencing), and mucus barrier function by *ex vivo* imaging were analysed.

**Results:**

Despite relatively small changes in biliary bile acid concentration and composition, more genes were differentially expressed in the colons of *Fxr*-livKO mice than in those of *Fxr*-intKO and *Fxr*-totKO mice (3272, 731, and 1824, respectively). The colons of *Fxr*-livKO showed increased expression of antimicrobial genes, Toll-like receptors, inflammasome-related genes and genes belonging to the ‘Mucin-type O-glycan biosynthesis’ pathway. *Fxr*-livKO mice have a microbiome profile favourable for the protective capacity of the mucus barrier. The thickness of the inner sterile mucus layer was increased and colitis symptoms reduced in *Fxr*-livKO mice.

**Conclusions:**

Targeting of *FXR* is at the forefront in the battle against metabolic diseases. We show that ablation of *Fxr* in the liver greatly impacts colonic gene expression and increased the colonic mucus barrier. Increasing the mucus barrier is of utmost importance to battle intestinal diseases such as inflammatory bowel disease, and we show that this might be done by antagonising *FXR* in the liver.

**Lay summary:**

This study shows that the communication of the liver to the intestine is crucial for intestinal health. Bile acids are key players in this liver-to-gut communication, and when *Fxr*, the master regulator of bile acid homoeostasis, is ablated in the liver, colonic gene expression is largely affected, and the protective capacity of the mucus barrier is increased.

## Highlights

• *Fxr* ablation in the mouse liver has a major impact on colonic gene expression.
• *Fxr* signalling is induced in the colons of liver *Fxr* knockout (*Fxr*-livKO) mice.
• In *Fxr*-livKO colons, expression of antimicrobial and mucus genes is increased.
• Microbiome of *Fxr*-livKO mice is indicative of enhanced mucus barrier function.
• *Fxr*-livKO mice have an increased mucus barrier.

## Graphical abstract

**Figure:**
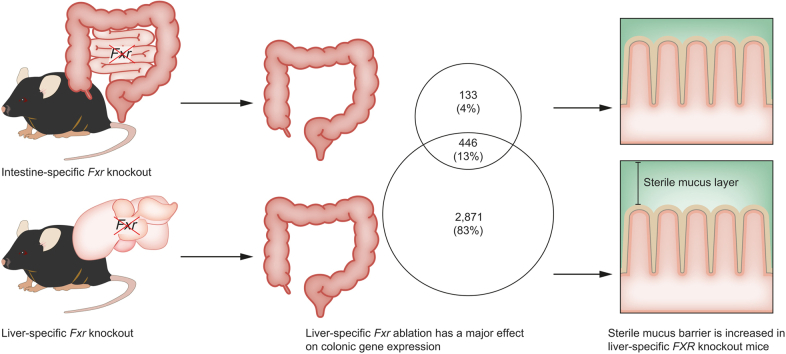

## Introduction

The gut–liver axis refers to the crosstalk between the intestine and the liver, particularly mediated by microbiome- and diet-derived metabolites. The (patho)physiological relevance of this crosstalk is increasingly recognised. Key in this crosstalk are bile acids (BAs), which circulate between these organs within the enterohepatic circulation through the portal venous (gut-to-liver) and biliary (liver-to-gut) systems. In the liver, BAs are synthesised and conjugated with glycine or taurine, which increase their solubility. BAs are subsequently stored in the gallbladder. After food intake, bile is released into the intestine to facilitate absorption of dietary lipids. The majority (∼95%) of BAs is reabsorbed in the ileum and reaches the liver via the portal vein. Unabsorbed BAs enter the colon and are subsequently deconjugated and converted by bacteria into secondary BAs.^1^

Farnesoid X receptor (*Fxr*), which is the key regulator of BA transport, signalling, and metabolism^2^ is highly expressed in the gut–liver axis. In the liver, *Fxr* determines the synthesis of the primary BAs and their transport from liver cells into the biliary system by regulating transcription of several key genes, including *Cyp7a1*, *Cyp8b1*, *Shp*, and *Bsep.*^1^ In the ileum, where *Fxr* is activated by absorbed BAs, *Fxr* regulates transcription of *Ibabp*, *Ostα*, and *β*, which are involved in BA uptake,^1^ and of *FGF15* (*FGF19* in humans), which is a key enterokine involved in gut–liver communication.^3^

Fgf15/19 is secreted at the basolateral membrane of enterocytes and reaches the liver via the portal vein. Here it binds to the Fibroblast growth factor receptor 4 (Fgfr4)/β-klotho complex on hepatocytes, which subsequently represses BA synthesis via *Cyp7a1*.^3^ Next to regulation of BA synthesis, *FXR*-mediated expression of *FGF15/19* has other functions. Mice lacking *Fgf15* fail to maintain glucose homoeostasis.^4^ Pharmacological administration or overexpression of *FGF19* restores glucose homoeostasis and glycogen metabolism.^5^^,^^6^ In conclusion, *FXR*-mediated *FGF15/19* expression plays a key role in gut-to-liver communication.

By contrast to gut-to-liver communication, much less is known about liver-to-gut communication. It is known that decreased bile flow from the liver to the intestine in humans and mice results in bacterial overgrowth, which is reversed by oral BA treatment.^7^^,^^8^ Additionally, BAs contribute to the exclusion of certain bacteria, favouring others.^7^^,^^8^ Some pathobionts, such as *Bilophila wadsworthia*, are not only BA resistant, but their growth is favoured by taurine-conjugated, but not glycine-conjugated, BA,^9^ indicating that hepatic BA output influences the gut microbiota composition, potentially impacting intestinal functions. However, the role of liver *Fxr* in this respect, for example, by modulating biliary BA concentration and composition, as well as the potential consequences on intestinal integrity and function remain elusive.

To gain a better understanding of liver-to-gut communication by *Fxr*-regulated BA synthesis and secretion, we generated mice in which we ablated *Fxr* tissue specifically in either the liver or the intestine and studied colonic gene expression, the microbiome and mucus barrier characteristics.

## Materials and methods

### Mice

The experiment was approved by the ethics committee of the University Medical Center Utrecht and was in accordance with European law. To generate whole-body (*Fxr*-totKO), liver-specific (*Fxr*-livKO), and intestine-specific (*Fxr*-intKO) *Fxr*-null mice, homozygous *Fxr*-floxed mice (C57Bl6J *Fxr* fl/fl, kindly provided by K. Schoonjans, Ecole Polytechnique Federale de Lausanne, Switzerland^10^) were crossed with C57Bl6J mice harbouring a cre-recombinase allele under the control of the meox2 promoter (Meox2-cre mice, stock 003755), albumin promoter (Alb-Cre mice, stock 003574), and villin promoter (Villin-Cre mice, stock 004586) from The Jackson Laboratory, respectively. *Fxr*-floxed mice were used as control. For genotyping, DNA was isolated from the mouse ear. Genotyping was performed as described previously^10^ and by protocols supplied by Jackson Laboratory. Ablation of *Fxr* mRNA in the colonic tissue was validated by real-time quantitative PCR (RT-qPCR) using primers Fw: 5′-tgagaacccacagcatttcg-3′, Rv: 5′-gcgtggtgatggttgaatgtc-3′. qPCR was performed on a CFX384 Real-Time system by using FastStart Universal SYBR Green Master Mix (Roche, Basel, Switzerland).

Male mice of 9–12 weeks old were included. The number of mice used per experiment is indicated in the figure legends. Mice were fed a purified diet (AIN-93M, Research Diets, Wijk bij Duurstede, The Netherlands) and drinking water *ad libitum*. Mice were individually housed in a room with controlled temperature (20–24°C), relative humidity (55% ± 15%), and a 12-h light–dark cycle. Mice were acclimatised for 1 week; after that, the intervention period of 1 week started in which mice remained on the same diet and body weight was recorded. Mice were fasted 4 h before sacrifice, and 0.6 mg/g bodyweight of fluorescein isothiocyanate (FITC)-conjugated dextran (Sigma, St. Louis, MO, USA; molecular mass 3–5 kDa) was given to measure intestinal permeability, which is a measurement to determine epithelial leakage by dysfunctional tight junctions. After 4 h, blood was collected to measure FITC plasma concentrations. The colon was excised, mesenteric fat was removed, and the colon was opened longitudinally, washed in PBS, and cut into 3 parts. The middle 1.5-cm colon tissue was formalin fixed and paraffin embedded for histology. The remaining proximal and distal parts were scraped. Scrapings include the epithelial lining and lamina propria, but not the muscle layer. These scrapings were pooled per mouse, snap-frozen in liquid nitrogen, and stored at −80°C until further analysis. Colonic contents were sampled and snap-frozen for microbiota analysis. Colitis was induced by administration of 2.5% (w/v) dextran sodium sulfate (DSS; molecular mass 36–50 kDa; MP Biomedicals, Solon, Ohio, USA) in drinking water for 10 days. Rectal bleeding was scored on a scale from 0 to 5, indicating no (0) to very severe (5) rectal bleeding.

### Western blot analysis

Western blot on the liver, colon, and kidney tissue was performed as described in the Supplementary information.

### RNA isolation, sequencing, and qPCR

Total RNA from the colon was isolated for sequencing and qPCR; see Supplementary information and File S1. Sequencing data can be found in Gene Expression Omnibus under accession number GSE163157.

### Histology and immunohistochemistry

H&E, Muc2, and high-iron diamine (HID) stainings and quantification are described in the Supplementary information.

### Plasma and gall bladder BAs

Plasma and gall bladder BAs were determined as described in the Supplementary information.

### Bacterial DNA isolation and 16S rRNA gene sequencing and analyses

Bacterial DNA was isolated for 16S rRNA gene sequencing, as described in the Supplementary information.

### Mucus layer measurements

Mucus barrier function was determined in n = 5 *Fxr*-intKO and *Fxr*-livKO and n = 7 control mice. See the Supplementary information.

### Statistical analysis

Differences between the groups were tested by Student’s *t* test using GraphPad Prism 8 (GraphPad Software, San Diego, California, USA). RNA and bacterial sequencing data were analysed statistically as indicated in the respective methods descriptions.

## Results

**Fig. 1:**
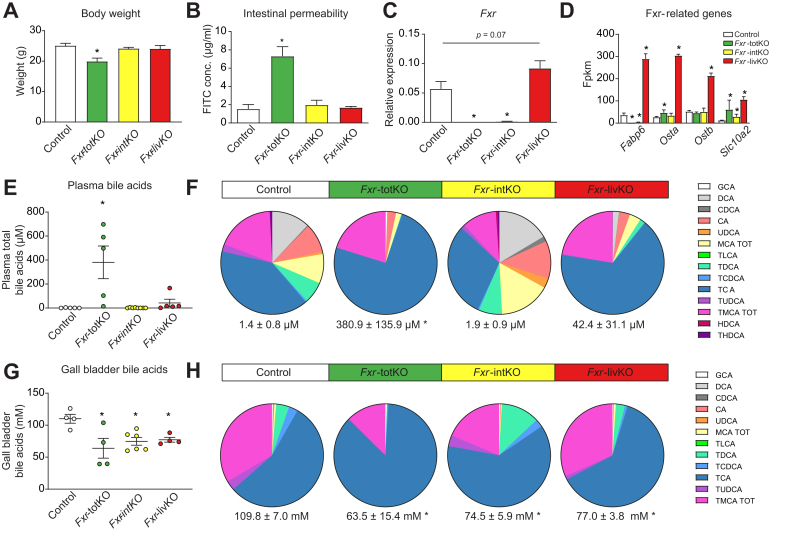
Characteristics of the animal models. (A) Body weight of the different models. (B) Intestinal permeability as determined by FITC dextran plasma concentrations. (C) Relative FXR expression in colon determined by qPCR. (D) Expression of *Fxr*-related genes in fpkm as determined by RNA sequencing. (E) Plasma bile concentrations and (F) relative plasma bile composition. (G) Gall bladder bile concentration and (H) relative gall bladder bile composition. All data are shown as mean ± SEM (n = 4–8 mice per group, except for (D) in which n = 3). Values for *Fxr*-intKO, *Fxr*-livKO, and *Fxr*-totKO *vs.* controls were tested using Student’s *t* test, and ∗ indicates *p* <0.05. FITC, fluorescein isothiocyanate; fpkm, fragments per kilobase of transcript per million mapped reads; FXR, farnesoid X receptor; *Fxr*-intKO, intestine-specific *Fxr* knockout; *Fxr*-livKO, liver-specific *Fxr* knockout; *Fxr*-totKO, whole-body *Fxr* knockout; qPCR, quantitative PCR.

To study the *Fxr*-dependent communication between the liver and the intestine, we compared *Fxr* floxed (control) with whole-body (*Fxr*-totKO), intestine-specific (*Fxr*-intKO), and liver-specific (*Fxr*-livKO) *Fxr* knockout mice. Absence of *Fxr* protein was validated by Western blot (Fig. S1). Ablation of *Fxr* in either the liver or the intestine had no effect on body weight (Fig. 1A) or intestinal permeability (Fig. 1B). However, the *Fxr*-totKO mice had a significantly decreased body weight and an increased intestinal permeability. Ablation of *Fxr* in the colons of the *Fxr*-intKO and *Fxr*-totKO was checked by qPCR (Fig. 1C). Remarkably, *Fxr* expression in colonocytes of the *Fxr*-livKO tended to increase. Colon-specific *Fxr-*target genes are currently unidentified, but we see a significant increase of known ileal *Fxr*-target genes in the colons of the *Fxr*-livKO (Fig. 1D), implying that *Fxr* signalling is increased in colonocytes of the *Fxr*-livKO mice.

Because *Fxr* is the main regulator of BA synthesis, we investigated plasma and gall bladder BA levels and composition. In plasma, the serum BA concentration was not different between the *Fxr*-livKO, *Fxr*-intKO, and control mice (Fig. 1E). This is in line with previous data,^11^ showing that BA levels can be maintained when *Fxr* is ablated in either the liver or the intestine. The total BA concentration was strongly increased in the *Fxr*-totKO, mainly caused by elevated levels of conjugated BA (Fig. 1F), which indicates that this model has severe liver problems leading to spillover of BA to the systemic circulation. A previous study also found increased plasma bile concentrations in the *Fxr*-totKO, but not in the *Fxr*-livKO and *Fxr*-intKO,^12^ although the total plasma concentration was lower, which might be explained by a different diet or the difference in the Fxr KO model (exon 11 *vs.* exon 6 ablation).

The total BA concentration in the gall bladder bile, which enters the intestine after gall bladder contraction, was decreased in the *Fxr*-totKO, *Fxr*-intKO, and *Fxr*-livKO compared with that in the controls (Fig. 1G). However, the relative composition of the gall bladder bile was not different between the *Fxr*-livKO and the controls (Fig. 1H). Overall, this showed that BA concentration and composition can be maintained in the absence of *Fxr* in either the liver or the intestine. The ablation of FXR in both tissues, or the accompanying ablation of FXR in other tissues in the *Fxr*-totKO, causes the deregulation of BA homoeostasis.

### Ablation of liver *Fxr* has a major impact on colonic gene expression

**Fig. 2:**
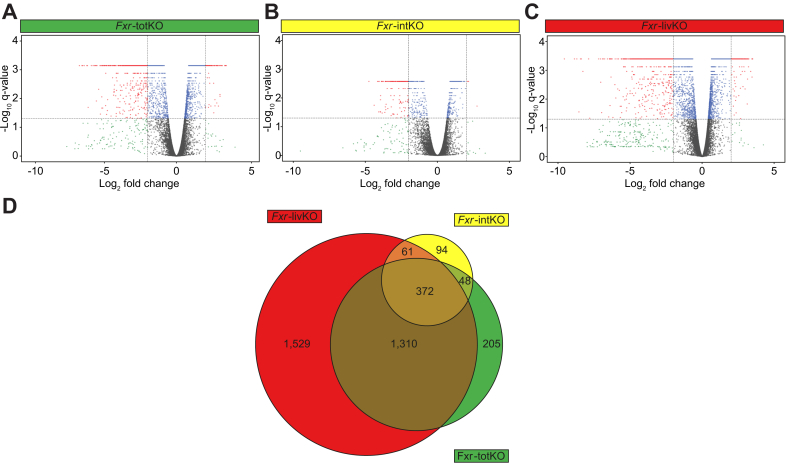
Ablation of *Fxr* in the liver has a major impact on colonic gene expression. Volcano plots of RNA sequencing data of colons of (A) *Fxr*-totKO, (B) *Fxr*-intKO, and (C) *Fxr*-livKO mice compared with controls. X-axes: genes located on the right-hand side of zero have a higher expression in the KO model compared with control, whereas points on the left of zero are more highly expressed in the control. Grey: |fold change| <2, q-value >0.05. Green: |fold change| >2, q-value >0.05. Blue: |fold change| <2, q-value <0.05. Red: |fold change| >2, q-value <0.05. The q-value is calculated by cufflink test statistics. (D) Proportional Venn diagram showing numbers of significantly changed genes in the colon of the different KO models compared with controls. *Fxr*, farnesoid X receptor; *Fxr*-intKO, intestine-specific *Fxr* knockout; *Fxr*-livKO, liver-specific *Fxr* knockout; *Fxr*-totKO, whole-body *Fxr* knockout; KO, knockout.

Next, we used RNA sequencing to investigate the effect of *Fxr* ablation on colonic gene expression. A distribution of the significant changes in expression for the *Fxr*-totKO, *Fxr*-intKO, and *Fxr*-livKO compared with the controls is visualised in volcano plots (Fig. 2A–C). The *Fxr*-livKO group shows the largest number of differentially expressed genes as well as the highest log2 fold changes (Fig. 2C). Remarkably, the effect of ablating *Fxr* in the liver on colonic gene expression is much larger than the effect of intestine-specific ablation or whole-body *Fxr* ablation. The Venn diagram (Fig. 2D) illustrates that the colons of the *Fxr*-livKO show in total 3,272 differentially expressed genes, of which 47% is specific for this model and not shared by the *Fxr*-totKO and *Fxr*-intKO. Ablation of liver *Fxr* is thus a major determinant of colonic gene expression, underlying the importance of liver-to-gut communication.

### Ablation of liver *Fxr* changes colonic defence response to bacteria

**Fig. 3:**
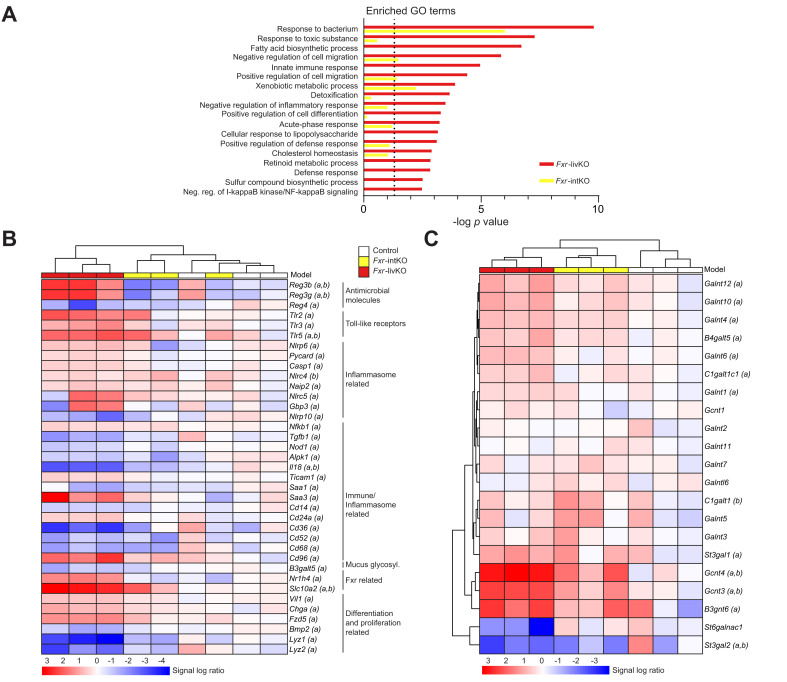
GO-term analysis shows enrichment for ‘response to bacterium’ in the *Fxr*-livKO. (A) GO terms of interest that were enriched in *Fxr-*livKO. Bars show the −log_10_ of the Fisher’s exact test *p* value from the GO enrichment in *Fxr-*livKO (red) and *Fxr-*intKO (yellow). Vertical dotted line represents the significant *p* value cut-off of 0.05 (1.3 on the −log_10_ scale). Analysis included RNA sequencing data of n = 3 mice per group. (B) Heatmap of the normalised expression per sample of a selection of genes belonging to the GO term ‘response to bacterium’ (GO:0009617, unsupervised clustering of the groups). N = 3 mice per group. (C) Heatmap of genes belonging to the KEGG pathway ‘Mucin type O-glycan biosynthesis’ (mmu00512) (unsupervised clustering). In brackets behind the gene name, significance is indicated with ‘a’ meaning different in *Fxr-*livKO compared with controls and ‘b’ indicating a difference in *Fxr-*intKO compared with controls (q <0.05). The average expression for the controls is set to 1. N = 3 mice per group. *Fxr*, farnesoid X receptor; *Fxr*-intKO, intestine-specific *Fxr* knockout; *Fxr*-livKO, liver-specific *Fxr* knockout; GO, Gene Ontology; KEGG, Kyoto Encyclopedia of Genes and Genomes.

To get more insight into the affected pathways in the colons of the *Fxr*-livKO and *Fxr*-intKO compared with the controls, we performed Gene Ontology (GO)-term enrichment analysis. For reasons indicated above, the *Fxr*-totKO group was excluded from further analysis. The main GO categories that were enriched in the *Fxr*-livKO compared with the controls involved defence response to bacteria, metabolism, and inflammation (Fig. 3A). All these pathways were regulated to a lower extent or not significant in the *Fxr*-intKO compared with the controls. With regard to the GO term ‘response to bacterium’, 165 out of 367 genes were significantly different in the *Fxr*-livKO *vs.* controls, compared with only 31 in the *Fxr*-intKO. We performed hierarchical clustering on all genes included in the ‘response to bacterium’ GO term (GO: 0009617) (Fig. 3B and qPCR validation in Fig. S2). The controls and *Fxr*-intKO cluster separately from the *Fxr*-livKO. Remarkably, the antimicrobial molecules regenerating islet-derived 3 beta and gamma (*Reg3β* and *Reg3γ*, respectively) were increased in the *Fxr*-livKO but decreased in the *Fxr*-intKO compared with the controls. The expression of *Reg3β* and *Reg3γ* is stimulated by activation of Toll-like receptors (*Tlrs*). Notably, *Tlr2, Tlr3,* and *Tlr5* are upregulated specifically in the *Fxr*-livKO. *Nlrp6*, *Casp1*, and *Pycard* are inflammasome-related genes activated in the *Fxr*-livKO, and this Nlrp6 inflammasome is involved in goblet cell mucus secretion.^13^ Another mucus-related gene present in this GO term and decreased in the *Fxr*-livKO was *B3galt5*. Furthermore, several cell-type markers and immune-related genes were regulated.

Overall, genes involved in the defence against and (immunological) response to bacteria were altered in the *Fxr*-livKO, hinting towards possible differences in mucus characteristics. Genes belonging to the Kyoto Encyclopedia of Genes and Genomes (KEGG) pathway Mucin-type O-glycan biosynthesis (mmu00512) were visualised in a heatmap (Fig. 3C and qPCR validation in Fig. S2), and 12 out of 21 identified genes belonging to this pathway were significantly different in the *Fxr-*livKO. Therefore, mucus glycosylation is likely altered in the *Fxr-*livKO*,* with potential effects on the gut microbiome composition and mucus barrier characteristics (as reviewed by Corfield^14^).

### *Fxr*-livKO have a microbiome composition with possible beneficial effects on the mucus barrier

**Fig. 4:**
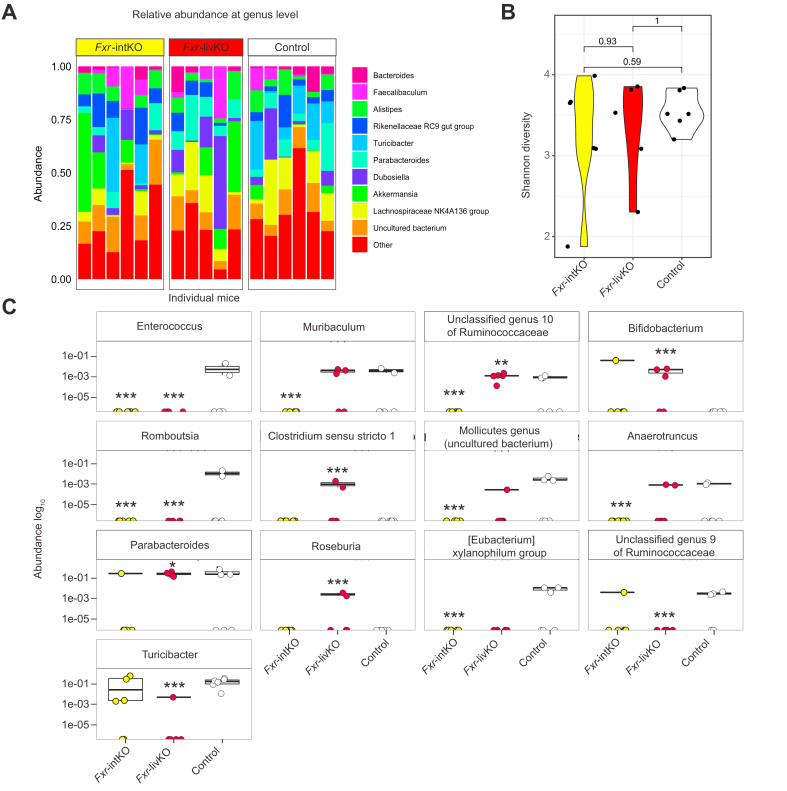
*Fxr*-livKO have a microbial profile indicative of increased mucus barrier integrity. (A) Relative abundance of microbiota at the genus level in individual samples (mice were individually housed). Depicted are the 10 most abundant genera. (B) Shannon diversity index (n = 5 for controls; n = 6 for *Fxr*-intKO and *Fxr*-livKO). (C) Bacteria genera that show statistically significant differences in relative abundance, as analysed with the statistical framework ANCOM-BC (Analysis of Compositions of Mircobiomes with Bias Correction) (n = 5 for controls; n=6 for *Fxr*-intKO and *Fxr*-livKO). Y-axis show the log_10_ relative abundance. ∗, ∗∗, and ∗∗∗ indicate *p* <0.05. *p* <0.01, and *p* <0.001, respectively. *Fxr*, farnesoid X receptor; *Fxr*-intKO, intestine-specific *Fxr* knockout; *Fxr*-livKO, liver-specific *Fxr* knockout.

The gut microbiome of the *Fxr*-intKO and *Fxr*-livKO was investigated by 16s sequencing and compared with that of the controls. The relative bacterial abundance per mouse for the 10 most abundant genera (Fig. 4A) showed that at a global level, microbial community structures were similar in all groups of mice. This is also apparent when examining the Shannon diversity index (Fig. 4B), which showed no significant differences between the *Fxr-*livKO and the *Fxr-*intKO compared with the controls. When differences in relative abundance for all genera present were analysed, a number of taxa were found to be either enriched or depleted in 1 or more of the groups (Fig. 4C). In the *Fxr*-livKO, the butyrate producer *Roseburia* is increased in abundance as well as *Bifidobacterium* and *Clostridium sensu stricto 1*, which are all described to be beneficial for the mucus layer.[15], [16], [17] In addition, in the *Fxr*-livKO, there was a decreased abundance of the genus *Turicibacter* and a member of the Ruminococcaceae family. Both taxa are predicted mucin degraders. A study on the degradation of human-synthetised mucin glycans and utilisation of the derived monosaccharides showed that 2 analysed *Turicibacter* genomes contained several glycosyl hydrolases, as well as catabolic pathways, for mucus-derived monosaccharides.^18^ Most of the same functions were also found in the known mucin degrader *Akkermansia muciniphila* (Table S2). Based on this information, we infer that *Turicibacter* is a mucus-degrading bacterium. Likewise, various members of the Ruminococcaceae family have been described to live in and degrade host mucin glycans.^19^ This information suggests that the microbiota of the *Fxr*-livKO mice contain a microbiota signature that may increase the protective capacity of the mucus barrier.

### *Fxr*-livKO have an increased mucus barrier

**Fig. 5:**
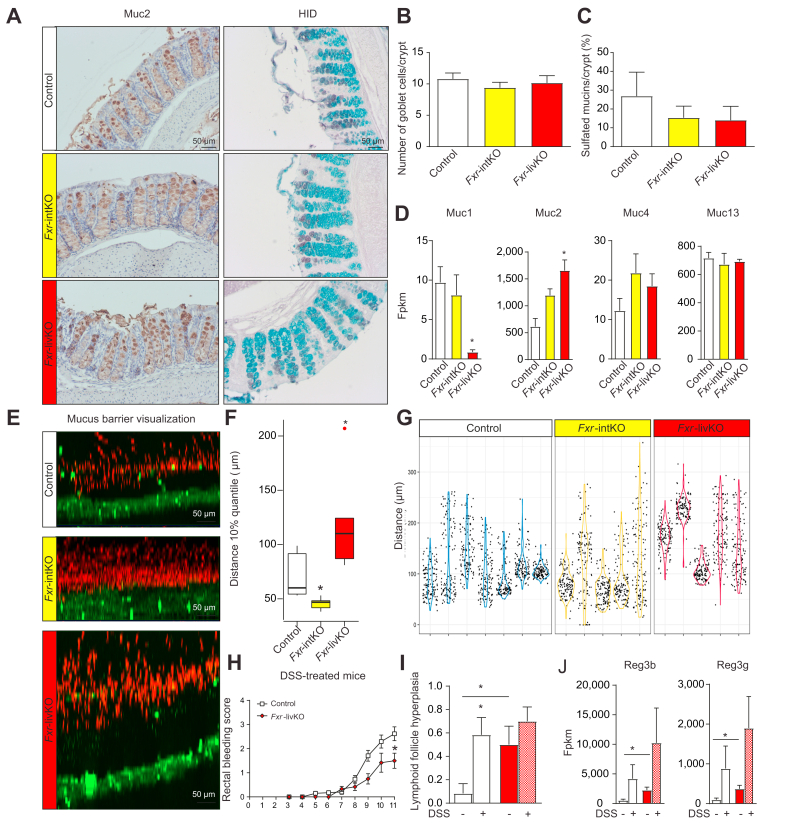
*Fxr*-livKO show an increased mucus barrier function. (A) Representative pictures of Muc2-staining and HID staining (sulfated mucins in brown and carboxylated mucins in blue). (B) Quantification of the number of Muc2-positive cells per crypt. Data are represented as mean ± SEM (n = 6 for controls, n = 7 for *Fxr*-intKO, and n = 5 for *Fxr*-livKO). Differences between *Fxr*-intKO and *Fxr*-livKO *vs.* controls were tested using Student’s *t* test, and ∗ indicates significant differences (*p* <0.05). (C) Quantification of the percentage of goblet cells filled with sulfated mucins per crypt. Data are represented as mean ± SEM (n = 5 for controls, n = 7 for *Fxr*-intKO, and n = 5 for *Fxr*-livKO). Differences between *Fxr*-intKO and *Fxr*-livKO *vs.* controls were tested using Student’s *t* test, and ∗ indicates significant differences (*p* <0.05). (D) Expression of the transmembrane mucins Muc1, Muc4, and Muc13 and the secreted mucin Muc2 in fpkm from RNA sequencing data (n = 3 per group). Differences between *Fxr*-intKO and *Fxr*-livKO *vs.* controls were tested using Student’s *t* test, and ∗ indicates significant differences (*p* <0.05). Expression levels of Muc5ac, Muc5b, Muc6, Muc15, Muc19, and Muc20 were also included in the RNA sequencing dataset; however, these were expressed at very low levels or completely absent and therefore not considered. (E) Permeability of the distal colonic mucus layer was visualised by adding 1-μm fluorescent beads (red) to the epithelial tissue (green). Shown are representative maximum projections of a resliced z-stack, showing a summarised view of the sample from the side. (F) The distance between beads and the epithelium was measured, and the 10% quantile, the closest to the epithelium, was depicted (average of n = 7 for controls, n = 5 for *Fxr*-intKO, and n = 5 for *Fxr*-livKO). Differences between *Fxr*-intKO and *Fxr*-livKO *vs.* controls were tested using Student’s *t* test, and ∗ indicates significant differences (*p* <0.05). (G) Violin plot of the distance of a random subset of 130 identified beads to the epithelial cells plotted for all the individual mice (n = 7 for controls, n = 5 for *Fxr*-intKO, and n = 5 for *Fxr*-livKO). (H) Rectal bleeding scores as marker for inflammation in DSS-treated mice (n = 8 controls and n = 6 for *Fxr*-livKO). Differences between *Fxr*-livKO *vs.* controls were tested per timepoint using Student’s *t* test, and ∗ indicates significant differences (*p* <0.05). (I) Lymphoid follicle hyperplasia scores in controls and *Fxr*-livKO with and without DSS treatment. Differences between *Fxr*-livKO and controls, and between with or without DSS within the animal model were tested using Student’s *t* test, and ∗ indicates significant differences (*p* <0.05). (J) Expression of Reg3β and Reg3γ in fpkm from RNA sequencing data (n = 3 per group). Differences between *Fxr*-livKO and controls and between with or without DSS within the animal model were tested using Student’s *t* test, and ∗ indicates significant differences (*p* <0.05). DSS, dextran sodium sulfate; fpkm, fragments per kilobase of transcript per million mapped reads; *Fxr*, farnesoid X receptor; *Fxr*-intKO, intestine-specific *Fxr* knockout; *Fxr*-livKO, liver-specific *Fxr* knockout; HID, high-iron diamine.

The mucus barrier quality is determined by mucus production in the goblet cells of the intestinal epithelium as well as by mucus breakdown at the luminal side by bacteria and proteases. Both the RNA sequencing data and the microbiome analysis point towards increased protective capacity of the mucus barrier of the *Fxr*-livKO. As a measure for mucus production, we first investigated the amount of goblet cells, by staining colon sections for Muc2 (Fig. 5A) and quantifying the number of goblet cells per crypt (Fig. 5B). There was no difference in the amount of goblet cells per crypt in the *Fxr*-intKO and *Fxr*-livKO compared with the controls. We performed HID staining (Fig. 5A) to investigate sulfation of mucins (brown). There was no difference in the percentage of sulfated mucins present in goblet cells (Fig. 5C). There was also no apparent change in filling of the goblet cells. Gene expression of Muc1 was decreased and that of Muc2 increased in the *Fxr-*livKO compared with the controls (Fig. 5D). To investigate differences in mucus barrier characteristics, we determined the mucus barrier permeability *ex vivo* by using upright microscopy and red fluorescent beads with the size of bacteria (1 μm).^20^ The distance between the beads and the colonic epithelium (in green) represents the thickness of the colonic inner impermeable mucus layer, which was increased in the *Fxr-*livKO compared with the controls and *Fxr*-intKO (Fig. 5E–G). This implies that the colonic epithelial cells of the *Fxr*-livKO are better protected against threats (*e.g.* bacteria and toxins) from the luminal side. This implication is in contrast to that for the *Fxr*-intKO, which have a more permeable and less protective mucus barrier. These data are in line with gene expression of the defence- and mucus-related genes (Fig. 3) and with the microbiota signature (Fig. 4) in the *Fxr*-livKO. We investigated if the *Fxr*-livKO are better protected against DSS-induced colitis. Rectal bleeding, a hallmark of inflammatory bowel disease (IBD), was lower in the *Fxr*-livKO than in the controls at Day 11 (Fig. 5H). Lymphoid follicle hyperplasia scores suggest that the *Fxr*-livKO have, next to a thicker mucus layer, a more active adaptive immune system. Expression levels of the protective *Reg3β* and *Reg3γ* were significantly increased at baseline and tended to increase even further upon DSS treatment in the *Fxr*-livKO compared with wild-type mice. Together, these data suggest the ablation of liver Fxr decreased the effects of DSS-induced colitis.

## Discussion

In this study, we aimed to get a better understanding of liver-to-gut communication mediated via *Fxr*. We show that ablation of *Fxr* in the liver is a major determinant of colonic gene expression and leads to an improved colonic mucus barrier function. This is in sharp contrast to *Fxr* ablation in the intestine, which resulted in worsening in mucus barrier function compared with the controls.

There are several reasons that might explain the major effect of liver *Fxr* ablation on the colon. Firstly, *Fxr-*target genes are induced in the colons of the *Fxr*-livKO, This upregulation is indicative of increased *Fxr* signalling in colonocytes of the *Fxr*-livKO, which might contribute to the changes in metabolic, inflammatory, and bacterial response pathways. Currently, we have no explanation for the upregulation of colonic *Fxr*-target genes caused by liver *Fxr* ablation, as we found no large differences in biliary BA concentration and composition in the *Fxr*-livKO compared with the controls. At this point, we cannot exclude that the total amount of bile secreted into the intestine and/or the spillover from the ileum to the colon in the *Fxr*-livKO is increased and may contribute to the upregulation of *Fxr*-target genes in the colons of these mice. Previously, increased plasma cholesterol levels were found in the *Fxr*-livKO,^21^ which might stimulate BA production.

Secondly, with regard to mucus synthesis, BAs have been shown to increase Cdx2 and Muc2 expression via the FXR/NF-κB signalling pathway in gastric epithelial cells.^22^ Cdx2 expression was increased 4-fold in the *Fxr*-livKO, as was the expression of the major secreted mucin Muc2 (3-fold). The absence of expression of the transmembrane mucin Muc1 is in line with the increased mucus barrier, as it was shown that Muc1 is upregulated under inflammatory conditions,^23^ and in diverse cancers, including breast, ovarian, lung, and colon cancer. Therefore, decreased Muc1 expression is suggestive of increased colon health. It is thought that Muc1, via its intracellular domain, stimulates various signalling pathways involved in cell survival through alterations of cell growth, proliferation, and cell death.^24^ Furthermore, Muc1 is a negative regulator of TLR signalling (reviewed in Dhar and McAuley^23^) and lower expression in the *Fxr*-LivKO coincides with increased expression of Tlr2, Tlr3, and Tlr5 in this model. We speculate that intestinal *Fxr* and *Fxr*-target genes play a role in the increased mucus barrier. Healthy mucus is glycosylated and often heavily sulfated to maintain barrier function by conferring resistance to bacterial enzymatic degradation. Decreased sulfation^25^ and decreased glycosylation^26^ is found in patients with IBD. The *Fxr*-livKO showed increased expression of several key glycosylating genes such as Gcnt3 and Gcnt4.

Importantly, *Reg3γ* and *Reg3β* were upregulated in the *Fxr*-livKO but downregulated in the *Fxr*-intKO. *Reg3β* and *Reg3γ* are antimicrobial molecules involved in maintaining distance between bacteria and the epithelial lining.[27], [28], [29] *Reg3γ* knockout mice display an altered mucus distribution, resulting in increased bacterial–epithelial contact, and increases in expression of innate immune response genes in the ileum,^30^ but not in the colon. Our data do show an association of the impermeable mucus thickness with the expression of *Reg3γ* in the colon of the mouse models. Furthermore, the *Fxr*-LivKO showed reduced rectal bleeding upon DSS administration, which was in line with increases in expression of the protective *Reg3γ* and *Reg3β*.

It has been shown that mucus secretion is regulated by the inflammasome.^13^ Deletion of NLRP6, and key components caspase 1 and ASC (*Pycard*) involved in inflammasome signalling, leads to the protrusion of mucus granules, which do not fuse with the apical basement membrane and release their mucins, but the entire cells are sloughed off into the lumen. We showed an increased expression of inflammasome-related genes in the *Fxr*-livKO, potentially stimulating mucus secretion. Lymphoid follicle hyperplasia scores suggest that the *Fxr*-livKO have, next to a thicker mucus layer, a more active adaptive immune system. Interestingly, it has recently been shown that the microbiome modulates adaptive immunity in mice by formation of secondary BA species that act on RORγ^+^ regulatory T cells via the vitamin D receptor, thereby lowering the vulnerability for chemically induced colitis.^31^

The microbiota signature that we found in the *Fxr*-livKO is supportive of an increased mucus barrier function, with several bacterial taxa present that might enforce the mucus barrier (*Roseburia*, *Bifidobacterium*, and *Clostridium sensu stricto 1*) and several taxa absent that might degrade mucins (*Turicibacter* and a member of the Ruminococcaceae family). Remarkably, in patients with IBD, the mucus barrier is often compromised,^32^ and decreased abundance of *Roseburia, Bifidobacterium*, and *Clostridium* are reported in (early-onset) IBD.^33^^,^^34^

Overall, we can conclude that ablation of *Fxr* in the liver has a major effect on colonic gene expression and improved mucus barrier characteristics. An impermeable inner mucus layer is important for the protection against diseases such as IBD.^32^ Therapeutic strategies to fortify the mucus barrier are therefore of utmost importance to battle IBD, and we show here the first indications that this may be done by antagonising FXR in the liver. Most likely, the increased *Fxr* signalling in the colonocytes of the *Fxr*-livKO contributes to this, as *Fxr* activation can inhibit inflammation and contributes to intestinal barrier preservation.^35^ As there are currently no liver-specific FXR antagonists available, giving an FXR antagonist to inhibit FXR function in the liver will also inhibit FXR function in the intestine, with possible consequences for the mucus barrier, and therefore, there is yet no therapeutic application for these new findings.

Targeting of FXR is currently at the forefront in the battle against metabolic diseases.^36^^,^^37^ In that respect, our study may imply that activation of FXR in the liver may have repercussions for mucus barrier integrity in the colon. Gastrointestinal problems have not been reported in clinical studies with FXR agonists, which may be because agonists simultaneously activate FXR in the intestine, inducing protection against intestinal inflammation.^35^ Together, we show that liver *Fxr* mediates colonic health, which, together with the already known communication from the intestine to the liver, points towards an FXR-dependent reciprocal communication between the liver and the intestine.

## Financial support

NI is supported by the MLDS Career Development grant (CDG16-04) and by the 10.13039/501100008955Wilhelmina Children’s Hospital Research Fund. NI and YV are supported by the Unusual Collaborations Grant from CUCo (Center for Unusual Collaborations; Strategic Alliance between Eindhoven University of Technology, Wageningen University & Research, Utrecht University, and the University Medical Center Utrecht). MRdZ is supported by a VIDI grant from the 10.13039/501100003246Netherlands Organization for Scientific Research (ZonMW, grant 917.15.377) and the Utrecht Exposome Hub of Utrecht Life Sciences. MB and YV are supported by NWO Gravitation 024.001.028. SvM is supported by the 10.13039/501100003246Netherlands Organization for Scientific Research (NWO) project ZonMW VICI (917.11.365) and project ZonMW Aspasia program (015.015.013).

## Authors’ contributions

Designed the study: NI, SvM. Interpreted the data: NI, KR, JP, SM, SvM. Conducted the animal experiment: NI, KR, EW. Conducted, analysed, and interpreted RNA sequencing data: JP, SM. Analysed and interpreted mucus barrier measurements: NI, MB, YV. Conducted bile acid measurements: CC, RP, FK. Performed, analysed, and interpreted bacterial sequencing data: MZ, SM, PS. Performed and analysed DSS colitis scoring: SY, AdB. Wrote the manuscript: NI. Critically revised the manuscript for important intellectual content: SvM, YV, FK.

## Data availability statement

Sequencing is submitted to Gene Expression Omnibus under accession number GSE163157. The 16S rRNA gene raw reads were deposited at the European Nucleotide Archive under the following project accession number: PRJEB47071.

## Conflicts of interest

Authors declare no conflict of interest, except for RP, CEO at TES Pharma; CC, employed by TESS Pharma; and SY, currently working at Janssen Pharmaceuticals.

Please refer to the accompanying ICMJE disclosure forms for further details.
