# Supplementary material for: Ablation of liver Fxr results in an increased colonic mucus barrier in mice
Source: JHEP Rep. 2021 Aug 4;3(5):100344. doi: 10.1016/j.jhepr.2021.100344 (PMC8463863; doi:10.1016/j.jhepr.2021.100344)
Supplement: Multimedia component 1 [file mmc1.pdf]

Ablation of liver *Fxr* results in an increased colonic mucus barrier in mice

Noortje Ijssennagger, Kristel S. van Rooijen, Stefanía Magnúsdóttir, José M. Ramos Pittol, Ellen C.L. Willemsen, Marcel R. de Zoete, Matthijs J.D. Baars, Paul B. Stege, Carolina Colliva, Roberto Pellicciari, Sameh A. Youssef, Alain de Bruin, Yvonne Vercoulen, Folkert Kuipers, Saskia W.C van Mil

Table of contents

Supplementary materials and methods....................3
Fig. S1....................10
Fig. S1....................11
Table S1....................12
Table S2....................13
Supplementary file 1....................14
Supplementary references....................18

Supplementary materials and methods

Western blot analysis

Homogenates of liver, colon and kidney tissue of *Fxr*-totKO, *Fxr*-livKO, *Fxr*-intKO and controls were made by adding 20µl of RIPA buffer containing protease inhibitors (Roche) per mg of tissue. Tissue was lysed using a tissue lyser (Qiagen). Lysates were spinned down at 14000 rpm for 10 min and supernatant was collected. Samples were incubated for 5 min at 95 °C with sample buffer. Samples were loaded on a 10% SDS PAGE gel and transferred on a nitrocellulose membrane, using a transfer buffer with 20% MetOH. Twenty micrograms total protein was loaded. The blot was probed overnight at 4 °C with an Anti-FXR Monoclonal antibody (A9033A, Invitrogen), and subsequently incubated with peroxidase-conjugated rabbit anti-mouse antibody (Agilent Dako, P0260) 1:10.000 for 1h at room temperature. Mouse-anti -β-actin -HRP (santa cruz, sc-47778)) was used as loading control. The signals were detected with the enhanced chemiluminescence detection system.

RNA isolation and sequencing

Total RNA from colon was isolated for sequencing and qPCR, using TRIzol reagent (Invitrogen) according to the manufacturer's protocol. For RNA sequencing, the RNA was further column purified using RNeasy Minikit columns (Qiagen).

mRNA sequencing libraries were generated with the NEXTflexTM Rapid RNA-seq Kit (Bio Scientific) using 1 µg total RNA. Sequencing was performed in the Nextseq500 platform (Illumina, Single-end, 75bp reads). Sequenced reads were aligned to the mouse reference genome mm9 using Tophat 2.18 [1]. Quantification of transcripts and differential expression were assessed using the Cufflinks suite

2.2.1 [2] using the Refseq mm9 gene annotation and default parameters. Differential gene expression was calculated pairwise between control mice (Fxr-fl/fl) and the KO models. Genes with a q-value lower than 0.05 were considered differentially expressed. All sequencing data and gene expression analysis has been deposited in the Gene Expression Omnibus under accession number GSE163157.

Gene Ontology (GO) enrichment analysis was done using the topGO [3] package in R [4] version 3.6.2. on differentially expressed genes. The mapping of gene identifiers was done using the genome wide annotation for mouse [5]) and the Affymetrix Human Genome U95 Set annotation data [6]. Enriched GO terms were identified using the topGO "weight01" algorithm in combination with the statistical methods Fisher's exact test and Kolmogorov–Smirnov test. Volcano plots were generated using the EnhancedVolano [7] package in R. Venn diagrams were created using the eulerr R package [8] online application (http://eulerr.co/). All scripts can be found in **Supplementary File 1**.

qPCR

RNA was reverse transcribed using the iScript cDNA Synthesis Kit (Bio-Rad Laboratories BV, Veenendaal, The Netherlands). Real-time PCR was carried out using FastStart Universal SYBR Green Master Mix (Roche) on a CFX 384 Bio-Rad thermal cycler (Bio-Rad). mRNA expression of genes of interest were normalized to Cyclophillin. Primers sequences are shown in Supplementary **Table 1**.

Histology and immunohistochemistry

H&E staining was performed to assess the morphology of the tissue. Details on slide preparation and scoring of lymphoid follicle hyperplasia can be found in [9].

Hyperplastic lymphoid follicles: 0; no hyperplastic lymphoid follicles; 1, one hyperplastic lymphoid follicle; 2, two hyperplastic lymphoid follicles; 3, three hyperplastic lymphoid follicles; 4, >3 hyperplastic lymphoid follicles.

To stain and quantify Muc2 positive goblet cells, paraffin embedded colon sections (5 µm) were deparaffinized and rehydrated in a series of graded alcohols. Sections were incubated for 15 min in 3% H2O2 in PBS to block endogenous peroxidase activity. Sections were placed in antigen retrieval solution (sodium citrate buffer, pH=6) and heated in a microwave oven for 5 min 700W followed by 20 min 500W, after which they were cooled to room temperature. Sections were blocked with 10% normal goat serum (Sigma-Aldrich Chemie) in PBS-Tween 20 (0.05% v/v) for 30 min. Sections were then incubated with 1:500 rabbit anti-mouse/human Muc2 antibody (NBP1-31231, Novus Biologicals, USA, CO) for 1h at room temperature. After washing, slides were incubated with the HRP-conjugated goat anti-rabbit antibody (31460 Thermofisher Scientific, USA), 1:200 for 30 min at room temperature. DAB substrate was used for HRP visualization and slides were counter stained with hematoxyline. Cells from 15 well-oriented crypts (longitudinal section) were counted per animal.

For the High Iron Diamine staining (HID, staining sulfated mucins (brown) and carboxylated mucins (blue)), deparaffinized sections were incubated overnight in diamine solution. Then sections were incubated with alcian blue (pH-2.5) for 30 min. Percentages of sulfated mucins were calculated. 15 well-oriented crypts (longitudinal sections) were scored per animal.

Plasma Bile Acids (BAs)

Plasma BAs were quantified using liquid chromatography-mass spectrometry [10]. A mixture of internal standards (isotopically labelled BAs) was added to 25 µl of

plasma. Samples were centrifuged at 15,800×*g* and supernatants were transferred and evaporated at 40 °C under a stream of N_2. Samples were reconstituted in 200 µl methanol:water (1:1), mixed and centrifuged at 1800×*g* for 3 min. The supernatant was filtered using a 0.2 µm spin-filter at 2000×*g* for 10 min. 10 µl of filtrates was injected into the LC–MS system, consisting of a Nexera X2 Ultra High Performance Liquid Chromatography system (SHIMADZU, Kyoto, Japan), coupled to a Sciex Qtrap 4500 MD triple quadrupole mass spectrometer (SCIEX, Framingham, MA, USA). BAs were separated with a ACQUITY UPLC BEH C18 Column (1,7 µm x 2,1 x 100 mm) equipped with a ACQUITY UPLC BEH C18 VanGuard Pre-Column (1,7 µm x 2,1 x 5 mm), (Waters, Milford, MA, USA). Data were analyzed with Analyst MD 1.6.2 software.

Gall bladder BAs

Gall bladder BAs were quantified using an Ultra Performance Liquid Chromatography-Mass Spectrometry system (UPLC-MS^2, Acquity H-Class Bio UPLC from Waters). Mouse bile samples were diluted to 1:100 and 1:1000 with ammonium acetate buffer 15 mM (pH = 8.0): (acetonitrile/methanol=75/25 v/v) = 50:50, v/v. A mixture of internal standards (6-Ethyl Cholic Acid and Tauro-6-Ethyl Cholic Acid) in methanol was added to the samples with a final concentration of 2.5 µM. 5 µl of sample was injected into the UPLC-ESI-MS^2 system. The UPLC is coupled to a XEVO TQD (Waters) triple quadrupole mass spectrometer, operating with negative ionization electrospray. BAs acquisition was performed in Multiple Reaction Monitoring mode (MRM). The analytical column was a UPLC CSH Phenyl-Hexyl Column (Waters) 1.7 µm, 2.1 × 75 mm protected by a guard column (Waters) 2.1 × 5 mm.

Bacterial DNA isolation and 16S rRNA Gene Sequencing and Analyses

To isolate bacterial DNA, colonic contents were mixed with 250 µl 20% SDS (Ambion), 500 ul PB Buffer (Qiagen PCR Purification kit), 250 µl zirconia/silica beads (Biospec, 0.1mm diameter) and 550 µl phenol:chloroform:isoamyl alcohol (25:24:1, pH 7.9) (Sigma), and homogenized by beat beating (2 min bead beating, 2 min on ice, 2 min bead beating, Biospec Mini-Beadbeater-96). Homogenates were centrifuged at 8000 x g for 3 min at room temperature after which the aqueous phase was removed and applied to a Qiagen PCR purification column. DNA was purified from the aqueous phase according to the manufacturer's instruction with the addition of an extra wash with 750 µl Buffer PE. 16S rRNA gene sequencing of the V4 region using the Illumina MiSeq Platform (Illumina, 2x250bp paired-end reads) was performed as described previously [11].

Analyses of 16S rRNA data were performed with the QIIME™ 2 microbial community analysis pipeline using DADA2 [12, 13]. The statistical framework Analysis of Composition of Microbiomes with Bias Correction (ANCOM-BC) [14] in combination with the phyloseq package (1.34.0) and microbiomeutilities: Utilities for Microbiome Analytics (Version 1.00.09) [https://microsud.github.io/microbiomeutilities/] was used for the statistical analyses of microbiota composition.

Mucus layer measurements

Mucus barrier function was determined in n=5 *Fxr*-intKO and *Fxr*-livKO and n=7 control mice, fed a 25kGy sterilized mouse breeding diet V1124 (Ssniff). Mice were sacrificed and a 1 cm piece of colonic tissue, 2 cm above the rectum, was dissected for mucus measurements. Tissue was prepared and mounted in a chamber as

described previously [11]. The tissue was visualized by adding Syto9 green fluorescent nucleic acid stain (ThermoFischer) at the apical side. After incubation for 10 minutes, the staining was removed and subsequently FluoSphere Crimson microbeads (1 µm, ThermoFischer) diluted 30 times in Krebs mannitol buffer was added to the tissue, to measure mucus penetrability. The beads were allowed to sediment onto the mucus surface for 5 min, then the mucosal surface was gently washed with Krebs mannitol to remove excess microbeads. The apical chamber compartment was then filled with 1 ml Krebs mannitol. Tissue and microbeads were visualized by acquiring Z-stacks on a Zeiss AxioImager Z1 (Carl Zeiss, Germany) upright stand using a 20x/0.5 NA water dipping objective. For the fluorescent excitation an X-Cite 120 illuminator (metal-halide lamp) was used in combination with Zeiss filter cubes for GFP (Ex 470/40, Em 540/50) and Rhodamine (Ex 546/12, Em 602,5/65). An Apotome2 was used for optical sectioning (combining 5 images for each plane in the acquired z-stack) to enhance SNR by reducing the background signal (scattered out-of-focus light) and improving axial resolution. Images were acquired with a Zeiss AxioCam HRm (503 mono) and processed using Zen 2.6 Pro software.

The thickness of the impermeable inner mucus layer was quantified since the inner sterile mucus layer is representative of the barrier function. The thickness of the inner mucus layer is represented by the distance of fluorescent beads to the cell monolayer and was quantified using Fiji [12]. First, beads were identified in a maximum projection along the Z-dimension. Beads were separated by the Watershed algorithm. The 'analyze particles' module was used to identify individual beads (size 10-150 pixels, circularity 30-100%, exclusion of beads at image edges). Bead XY-coordinates, and size were recorded. The bead Z-coordinates were

determined in a XZ resliced Z-stack of the beads channel. For each bead a sum projection was generated of XZ images containing the bead and a rectangular selection was made spanning the bead width and full Z range. The peak of signal intensity within this selection, along the Z axis, was used as the bead Z-coordinate. The cell monolayer was identified in a XZ resliced Z-stack of the cellular signal. This was performed by thresholding median projections of each 10 pixels in the Y-dimension. Saturated regions and floating cells were excluded from analysis using a binary filter reconstruct from the Morphology package (G. Landini Software). The distance of a bead to the monolayer was determined as the vertical distance between the bead Z-coordinate and the monolayer in the same XZ slice.

For comparison between the groups, the 10% quantile of distances of beads to the epithelial cells per mouse, was used and the differences between the groups were tested by a Students-T test using Graphpad Prism 8. For comparison of individual mice, a random subset of 130 beads was plotted per mouse.

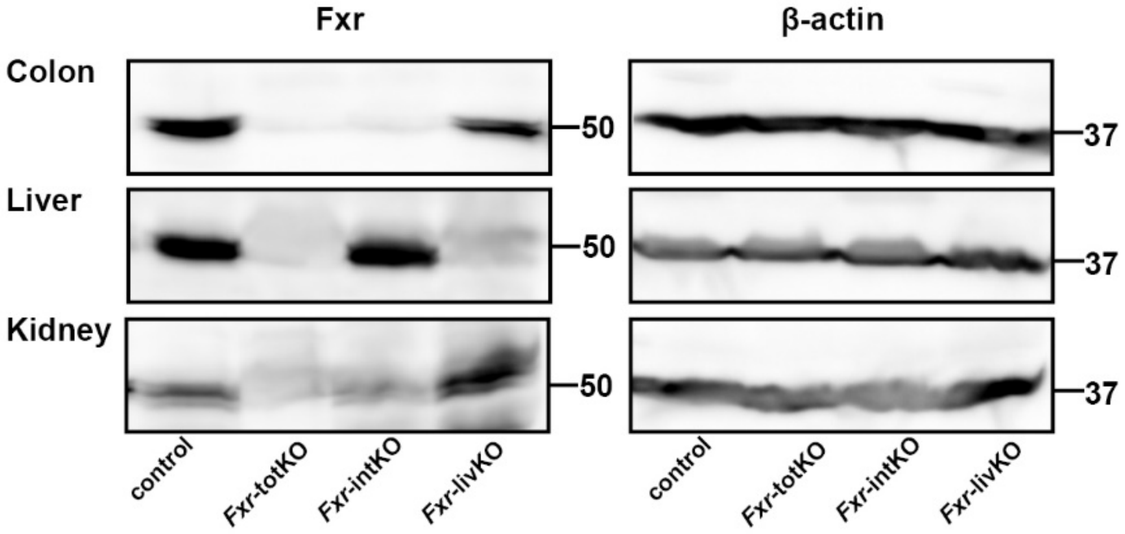

Fig. S1. Western blot for Fxr and β-actin in colon, liver and kidney of control, *Fxr*-totKO, *Fxr*-intKO and *Fxr*-livKO mice.

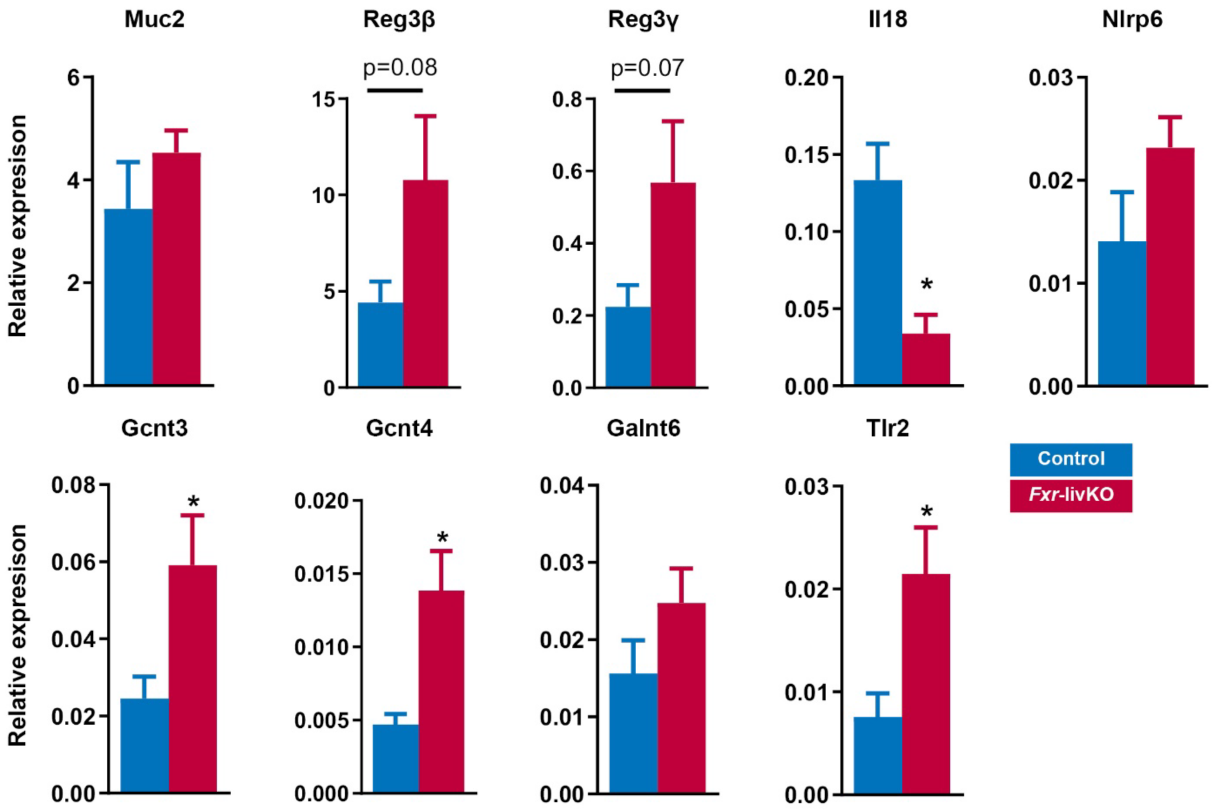

Fig. S2. qPCR validation of key genes. Data are represented as mean ± SEM (n=6 for controls, n=5 for *Fxr*-livKO). Differences between *Fxr*-intKO and *Fxr*-livKO versus controls were tested by Students T-test and * indicates significant differences ($P < 0.05$).

Table S1. Primer Sequences used for qPCR

Gene	Forward primer	Reverse primer
mCyclophillin	GGAGATGGCACAGGAGGAA	GCCCGTAGTGCTTCAGCTT
mFxr	ACAGCTAATGAGGACGACAG	GATTTCCTGAGGCATTCTCTG
mMuc2	ATGCCCACCTCCTCAAAGAC	GTAGTTTCCGTTGGAACAGTGAA
mReg3b	ATGCTGCTCTCCTGCCTGATG	CTAATGCGTGCGGAGGGTATATTC
mReg3g	GTATGGATTGGGCTCCATGA	GATTCGTCTCCCAGTTGATG
mIl18	GACTCTTGCGTCAACTTCAAGG	CAGGCTGTCTTTTGTCAACGA
mNlrp6	GGCAGCAGGTAAGCTATACCA	CGATTGGGTTGCGCCAGTA
mGcnt3	AGAGTTCCATCAACTGCTCAGG	TCTGCTGTCATCCTAAGGTAGTC
mGcnt4	TACGAGCACGAGCCTTTGG	AGGGTCTGATAAACGTCACAGT
mGalnt6	AGGTGCTGTGAACAACATTAGG	CCCAGAAGGGTTTCAGCTCAG
mTlr2	GGGGCTTCACTTCTCTGCTT	AGCATCCTCTGAGATTTGACG

Table S2. Glycosyl hydrolases in Akkermansia and Turicibacter

Glycosyl hydrolases							
**Genus/ species (# strains)**	**Alpha-N-acetyl galactosaminidases**	**Alpha-galactosidases**	**Alpha-L-fucosidases**	**Alpha-N-acetyl glucosaminidase**	**Beta-galactosidases**	**Beta-hexosaminidases**	**Sialidases**
Akkermansia muciniphila (1)		1	1	1	1	1	1
Turicibacter (2)		2	2		2	2	

Supplementary file 1. RNA-seq alignments to mouse reference genome mm9 using Tophat

```
# Download mm9 reference genome (https://hgdownload.soe.ucsc.edu/goldenPath/mm9/bigZips/)

# index mouse reference genome
bowtie2-build mm9.fa.gz mm9index

# align sample sequences to genome index
tophat2 -G mm9genes.gtf -o SAMPLE_ID mm9index
SAMPLE_ID_1.fastq.gz,SAMPLE_ID_2.fastq.gz,SAMPLE_ID_3.fastq.gz,SAMPLE_ID_4.fastq.gz

# Use cuffquant to quantify hits
cuffquant mm9genes.gtf ./SAMPLE_ID/accepted_hits.bam

# rename abundance file to include sample ID in file name
mv abundances.cxb SAMPLE_ID.cbx

# Differential expression using cufflinks
# RNA seq expression level files
# WT: 45.cxb,82.cxb,44.cxb
# intestinal KO: 75.cxb,15.cxb,13.cxb
# liver KO: 151.cxb,141.cxb,132.cxb
# total KO: 60.cxb,3.cxb,2.cxb

# differential expression example for WT vs. Intestinal KO
cuffdiff -o Wt-Intestine mm9genes.gtf 45.cxb,82.cxb,44.cxb 75.cxb,15.cxb,13.cxb

# open R
R

# load necessary packages in R
library(data.table)
library(ggplot2)
library(topGO)
library(org.Mm.eg.db)
library(hgu95av2.db)
library(pheatmap)
library(EnhancedVolcano)

# read in data
# liver KO data
liverKO <- fread("LiverKO-WT_gene_exp.diff")

# remove infinite fold changes
liverKO <- liverKO[is.finite(liverKO$`log2(fold_change)`),]

# only use results with status OK
liverKO <- liverKO[liverKO$status == "OK",]

# Volcano plot of differential expression
EnhancedVolcano(liverKO,
        lab = liverKO$gene,
        x = 'log2(fold_change)',
        y = 'q_value',
        pCutoff = 0.05,
        FCcutoff = 2,
        title = 'Liver KO vs. WT',
        subtitle = NULL,
        ylab = bquote(~-Log[10]~italic(q-value)),
```

```
            colAlpha = 1,
            transcriptPointSize = 1,
            gridlines.major = FALSE,
            gridlines.minor = FALSE,
            border = 'full',
            legend = c("ns","Log2 FC","q-value","q-value & Log2 FC"))
```

```
# Number of significantly different genes (q-value < 0.05) per KO model
signLiverKO <- liverKO[liverKO$q_value <= 0.05,]
signIntestinalKO <- intestinalKO[
            intestinalKO$q_value <= 0.05,]
signTotalKO <- totalKO[totalKO$q_value <= 0.05,]

# Get numbers for Venn diagram
# number of genes significiantly different in all KO models
length(intersect(signLiverKO$gene,
            intersect(signIntestinalKO$gene,  signTotalKO$gene)))
[1] 372
# number of genes significantly different in both liver and total KO, but not intestinal
length(setdiff(intersect(
            signLiverKO$gene, signTotalKO$gene),
            signIntestinalKO$gene))
[1] 1310
# number of genes significantly different in both liver and intestinal KO, but not total
length(setdiff(intersect(
            signLiverKO$gene, signIntestinalKO$gene),
            signTotalKO$gene))
[1] 61
# number of genes significantly different in both intestinal and total KO, but not liver
length(setdiff(intersect(
            signIntestinalKO$gene, signTotalKO$gene),
            signLiverKO$gene))
[1] 48
# Number of genes significantly different in liver but not intestinal or total
length(setdiff(signLiverKO$gene,
            union(signTotalKO$gene, signIntestinalKO$gene)))
[1] 1529
# Number of genes significantly different in intestinal but not liver or total
length(setdiff(signIntestinalKO$gene,
            union(signLiverKO$gene, signTotalKO$gene)))
[1] 94
# Number of genes significantly different in total but not liver or intestinal
length(setdiff(signTotalKO$gene,
            union(signLiverKO$gene, signIntestinalKO$gene)))
[1] 250
```

GO enrichment analysis

```
# define function to return top significant genes based on chosen cut-off of 0.05
topDiffGenes <- function(allScore) {
            return(allScore < 0.05)
}

# prepare liver KO data to test
geneListLiverKO <- liverKO$q_value
names(geneListLiverKO) <- liverKO$gene
sigGenesLiverKO <-
            names(geneListLiverKO)[topDiffGenes(geneListLiverKO)]

# GO enrichment liver
GO_L_BP <- new("topGOdata",
```

```
        description = "top GO in liver KO, BP",
        ontology = "BP",
        allGenes = geneListLiverKO,
        geneSel = topDiffGenes,
        nodeSize = 10,
        annot = annFUN.org,
        mapping = "org.Mm.eg.db",
        ID = "symbol")
```

```
# KS - often grabs more general GO terms
result_L_BP.ks <- runTest(GO_L_BP,
        algorithm = "weight01",
        statistic = "ks")
```

```
# Fisher's exact test - often grabs more specific/functional GO terms
result_L_BP.fisher <- runTest(GO_L_BP,
        algorithm = "weight01",
        statistic = "fisher")
```

```
# List both results in a table. Order by Fisher's test. Also show results for KS and the rank in KS.
allRes <- GenTable(GO_L_BP,
        KS = result_L_BP.ks,
        Fisher = result_L_BP.fisher,
        orderBy = "Fisher",
        ranksOf = "KS",
        topNodes = length(result_L_BP.ks@score),
        numChar=1000)
```

```
# Looking at genes in a specific GO term (or list of GO terms)
# select GO terms (comma separated list if multiple)
sel.terms <- c("GO:0009617")
```

```
# get the annotations
ann.genes <- genesInTerm(GO_L_BP, sel.terms)
```

```
# extract relevant genes from data set
sel.data <- liverKO[match(ann.genes$`GO:0009617`,
        liverKO$gene),]
```

```
# volcano plot of seleced genes related to GO term
EnhancedVolcano(
        sel.data,
        lab = sel.data$gene,
        selectLab = sel.data$gene,
        drawConnectors = TRUE,
        x = 'log2(fold_change)',
        y = 'q_value',
        pCutoff = 0.05,
        FCcutoff = 1,
        colAlpha = 1,
        transcriptPointSize = 2,
        title = "response to bacterium (GO:0009617)",
        subtitle = "LiverKO",
        ylab = bquote(~-Log[10]~italic(q-value)),
        gridlines.major = FALSE,
        gridlines.minor = FALSE,
        border = 'full',
        legend = c("ns","Log2 FC","q-value","q-value & Log2 FC"))
```

load source data
```
sourceDataTmp <-
fread("/Users/smagnusdottir/Projects/noortje_volcano/201113_source_data.csv", skip = 1)
```

normalize source data
```
sourceData <- t(as.matrix(sourceDataTmp[,
	2:ncol(sourceDataTmp)]))
sourceData <- as.data.frame(sourceData)
colnames(sourceData) <- sourceDataTmp$tracking_id
sourceData$samples <- rownames(sourceData)
sourceData$KOmodel <- c(
	"Fxr-intKO",
	"Fxr-intKO",
	"Fxr-intKO",
	"Fxr-totKO",
	"Fxr-totKO",
	"Fxr-totKO",
	"Fxr-livKO",
	"Fxr-livKO",
	"Fxr-livKO",
	"Control",
	"Control",
	"Control")
```

normalize data according to Noortje's formula
```
sourceData.num <- sourceData[, 1:23956]
sourceData.cat <- sourceData[, 23957:23958]
```

log2 of all signals
```
sourceData.num.log <- log2(sourceData.num)
```

average of all wt signals per column
```
wtMeans <- colMeans(
	sourceData.num.log[sourceData.cat$KOmodel == "WT",])
```

subtract average WT values from signals (log2)
```
sourceData.num.norm <- sourceData.num.log
for (i in 1:ncol(sourceData.num.log)){
	sourceData.num.norm[, i] <- sourceData.num.log[, i]
	 - wtMeans[i]
}
sourceData.num.norm <- t(sourceData.num.norm)
```

read in list of genes to plot in heatmap
```
fig2genes = fread("/Users/smagnusdottir/Projects/noortje_volcano/heatmap_fig_2b.csv", header =
FALSE)
```

```
# only WT, liver, and intestine
heatmap.data <-sourceData.num.norm[
	match(tolower(fig2genes$V1),
	tolower(rownames(sourceData.num.norm)))
	sourceData.cat$KOmodel %in% c("Control", "Fxr-intKO",
	"Fxr-livKO")]
```

define model types
```
koModels <- data.frame(model = sourceData.cat$KOmodel[sourceData.cat$KOmodel %in%
c("WT", "IntestinalKO", "LiverKO")])
rownames(koModels) <- colnames(heatmap.data)
```

define colors to match manuscript color palette

```
modelColors <- list(model = c(
	rgb(0/255, 115/255, 189/255), # Control
	rgb(255/255, 205/255, 52/255), # Fxr-intKO
	rgb(191/255, 0/255, 58/255)) # Fxr-livKO
)
names(modelColors$model) <- c("Control", "Fxr-intKO",
	"Fxr-livKO")

# set color palette for heatmap
paletteLength <- 50
myColor <- colorRampPalette(
	c("blue", "white", "red"))(paletteLength)
# define palette breaks based on data. Want max/min of data set to correspond to brightest red/blue colors
myBreaks <- c(seq(min(heatmap.data), 0,
	length.out = ceiling(paletteLength/2) + 1),
	seq(max(heatmap.data)/paletteLength, max(heatmap.data),
	length.out = floor(paletteLength/2)))

# create heatmap
pheatmap(heatmap.data,
	annotation_col = koModels,
	annotation_colors = modelColors,
	color = myColor,
	breaks = myBreaks,
	cluster_cols = TRUE,
	fontsize_row = 12)
```

Supplementary references

[1] Kim D, Pertea G, Trapnell C, Pimentel H, Kelley R, Salzberg SL. TopHat2: accurate alignment of transcriptomes in the presence of insertions, deletions and gene fusions. Genome Biol 2013;14.

[2] Trapnell C, Hendrickson DG, Sauvageau M, Goff L, Rinn JL, Pachter L. Differential analysis of gene regulation at transcript resolution with RNA-seq. Nat Biotechnol 2013;31:46-53.

[3] Alexa A, Rahnenfuhrer J. topGO: Enrichment Analysis for Gene Ontology. 2019.

[4] R Development Core Team. R: A language and environment for statistical computing. Vienna, Austria: R Foundation for Statistical Computing; 2019.

[5] Carlson M. org.Mm.eg.db: Genome wide annotation for Mouse. 2019.

[6] Carlson M. hgu95av2.db: Affymetrix Human Genome U95 Set annotation data (chip hgu95av2). 2016.

[7] Blighe K, Rana S, Lewis M. EnhancedVolcano: Publication-ready volcano plots with enhanced colouring and labeling. 2018.

[8] Larsson J. eulerr: Area-Proportional Euler and Venn Diagrams with Ellipses. 2020.

[9] Kojouharoff G, Hans W, Obermeier F, Mannel DN, Andus T, Scholmerich J, et al. Neutralization of tumour necrosis factor (TNF) but not of IL-1 reduces inflammation in chronic dextran sulphate sodium-induced colitis in mice. Clin Exp Immunol 1997;107:353-358.

[10] de Boer JF, Verkade E, Mulder NL, de Vries HD, Huijkman N, Koehorst M, et al. A human-like bile acid pool induced by deletion of hepatic Cyp2c70 modulates effects of FXR activation in mice. J Lipid Res 2020;61:291-305.

[11] Milona A, Owen BM, van Mil S, Dormann D, Mataki C, Boudjelal M, et al. The normal mechanisms of pregnancy-induced liver growth are not maintained in mice lacking the bile acid sensor Fxr. Am J Physiol Gastrointest Liver Physiol 2010;298:G151-158.

[12] Schindelin J, Arganda-Carreras I, Frise E, Kaynig V, Longair M, Pietzsch T, et al. Fiji: an open-source platform for biological-image analysis. Nature Methods 2012;9:676-682.
