# Supplementary material for: Ablation of liver Fxr results in an increased colonic mucus barrier in mice
Source: JHEP Rep. 2021 Aug 4;3(5):100344. doi: 10.1016/j.jhepr.2021.100344 (PMC8463863; doi:10.1016/j.jhepr.2021.100344)
Supplement: Multimedia component 2 [file mmc2.pdf]

JOURNAL
OF HEPATOLOGY

Journal of Hepatology

CTAT methods

Tables for a "Complete, Transparent, Accurate and Timely account" (CTAT) are now mandatory for all revised submissions. The aim is to enhance the reproducibility of methods.

- Only include the parts relevant to your study
- Refer to the CTAT in the main text as 'Supplementary CTAT Table'
- Do not add subheadings
- Add as many rows as needed to include all information
- Only include one item per row

If the CTAT form is not relevant to your study, please outline the reasons why:

1.1 Antibodies

Name	Citation	Supplier	Cat no.	Clone no.
Anti-FXR Monoclonal antibody		Invitrogen	A9033A	
peroxidase-conjugated rabbit anti-mouse antibody		Agilent Dako	P0260	
Mouse- anti -β-actin -HRP		Santa cruz	sc-47778	
rabbit anti-mouse/human Muc2 antibody		Novus Biologicals,	NBP1-31231	
HRP-conjugated goat anti-rabbit antibody		Thermofisher Scientific	31460	

1.2 Cell lines

Name	Citation	Supplier	Cat no.	Passage no.	Authentication test method

1.3 Organisms

Name	Citation	Supplier	Strain	Sex	Age	Overall n number
Fxr -floxed mice (C57Bl6J *Fxr* fl/fl,	[1])	kindly provided by K. Schoonjans, Ecole Polytechnique	C57Bl6J	M and F for breedings, M for experiment	9-12 weeks	13 in exp +8 for DSS treatment

		Federale de Lausanne, Switzerland [1])				
whole body *Fxr* -null mice (*Fxr*-totKO),		Crossing of floxed mice with Meox2-cre mice, stock 003755 Jackson Laboratory	C57Bl6J	M	9-12 weeks	8
intestine-specific *Fxr* -null mice (*Fxr*-intKO)		Crossing of floxed mice with Villin-Cre mice, stock 004586Jackson Laboratory	C57Bl6J	M	9-12 weeks	13
liver-specific *Fxr* -null mice (*Fxr*-livKO		Crossing of floxed mice with Alb-Cre mice, stock 003574 Jackson Laboratory	C57Bl6J	M	9-12 weeks	10 + 6 for DSS treatment

1.4 Sequence based reagents

Name	Sequence	Supplier

1.5 Biological samples

Description	Source	Identifier

1.6 Deposited data

Name of repository	Identifier	Link
Geo DataSets	GSE163157	https://www.ncbi.nlm.nih.gov/geo/query/acc.cgi?acc=GSE163157

1.7 Software

Software name	Manufacturer	Version
Graphpad Prism 8		
Fiji	[2].	

1.8 Other (e.g. drugs, proteins, vectors etc.)

1.9 Please provide the details of the corresponding methods author for the manuscript:

S.W.C van Mil
UMC Utrecht
Dept of Molecular Cancer Research, Center for Molecular Medicine
PO Box 85060
3508 AB Utrecht
The Netherlands
+31-(0)887550005
S.W.C.vanmil@umcutrecht.nl

2.0 Please confirm for randomised controlled trials all versions of the clinical protocol are included in the submission. These will be published online as supplementary information.

Not applicable

References

[1] Milona A, Owen BM, van Mil S, Dormann D, Mataki C, Boudjelal M, et al. The normal mechanisms of pregnancy-induced liver growth are not maintained in mice lacking the bile acid sensor Fxr. Am J Physiol Gastrointest Liver Physiol 2010;298:G151-158.
[2] Schindelin J, Arganda-Carreras I, Frise E, Kaynig V, Longair M, Pietzsch T, et al. Fiji: an open-source platform for biological-image analysis. Nature Methods 2012;9:676-682.
